# Mechanical circulatory support devices in heart failure: a narrative review of current evidence, clinical applications, complications, and future directions

**DOI:** 10.3389/fcvm.2026.1859170

**Published:** 2026-09-29

**Authors:** Ruirui Zhu, Fangyuan Zhang, Yudong Peng, Yucheng Zhong

**Affiliations:** 1 Department of Cardiology, Union Hospital, Tongji Medical College, Huazhong University of Science and Technology, Wuhan, China; 2 Hubei Key Laboratory of Biological Targeted Therapy, Union Hospital, Tongji Medical College, Huazhong University of Science and Technology, Wuhan, China; 3 Hubei Provincial Engineering Research Center of Immunological Diagnosis and Therapy for Cardiovascular Diseases, Union Hospital, Tongji Medical College, Huazhong University of Science and Technology, Wuhan, China; 4 Key Laboratory of Biological Targeted Therapy, (Huazhong University of Science and Technology), Ministry of Education, Wuhan, China; 5 Department of Dermatology, Union Hospital, Tongji Medical College, Huazhong University of Science and Technology, Wuhan, China; 6 Department of Cardiovascular Surgery, Union Hospital, Tongji Medical College, Huazhong University of Science and Technology, Wuhan, China; 7 Department of Cardiology, The People’s Hospital of Guangxi Zhuang Autonomous Region, and Institute of Cardiovascular Diseases, Guangxi Academy of Medical Sciences, Nanning, China

**Keywords:** advanced heart failure, bridge-to-transplant, cardiogenic shock, destination therapy, extracorporeal membrane oxygenation, impella, mechanical circulatory support devices, total artificial heart

## Abstract

Mechanical circulatory support devices (MCSDs) have evolved from rescue therapy for refractory cardiogenic shock (CS) to an essential component of advanced heart failure management, spanning temporary support, bridge strategies, durable left ventricular assist device (LVAD), and total artificial heart (TAH). This structured narrative review summarizes the historical evolution, current indications, device classification, major short-term and long-term platforms, adverse events, and future directions of MCSDs. Literature was selected in a structured narrative manner with emphasis on contemporary guidelines, randomized or comparative studies, major registries, and representative Chinese experience. Available evidence shows that MCSDs can improve hemodynamics and, in selected populations, survival and quality of life; however, these benefits must be balanced against bleeding, stroke, infection, right heart failure, device malfunction, cost, and major ethical and access constraints. Current practice thus necessitates careful patient selection, stage-based therapeutic escalation, and rigorous evaluation of mixed evidence pertaining to temporary MCSDs, LVAD and TAH, instead of merely describing individual devices.

## 1 Introduction

Heart failure (HF) is a major global public health problem and remains associated with substantial mortality, recurrent hospitalization, impaired functional status, and high economic burden (1). Advanced HF represents the end stage of multiple structural and functional cardiac disorders and is characterized by persistent symptoms, frequent decompensation, and progressive end-organ dysfunction despite guideline-directed therapy. In China, as in many other countries, population aging and improved survival from other cardiovascular diseases are increasing the number of patients who may ultimately require advanced therapies (2). Heart transplantation remains the gold-standard treatment for selected patients with end-stage HF, but donor shortage, waiting-list mortality, and contraindications continue to limit access.

MCSDs are advanced cardiac assist technology designed to partially or completely replace the function of a failing heart through mechanical means, thereby maintaining blood circulation and improving systemic hypoxia (3). Since their initial clinical application in the 1950s, MCSDs have undergone decades of evolution and development, and have now become a key therapeutic tool for patients with critical cardiac conditions and end-stage HF. These devices can not only provide effective hemodynamic support in the short term to help patients safely survive the critical phase, but also serve as a bridge-to-transplant therapy (BTT), offering a long-term MCSDs option for patients who are unable to undergo immediate heart transplantation.

With the increasing global burden of advanced HF, MCSDs have demonstrated important value in stabilizing hemodynamics, facilitating organ recovery, bridging selected patients to transplant or decision, and improving survival and quality of life in appropriately chosen candidates. At the same time, the field is constrained by bleeding, stroke, infection, right heart failure, aortic insufficiency, cost, and ethical concerns related to frailty and futility. Accordingly, a contemporary review should not only summarize device development, but also critically address the quality and heterogeneity of the evidence supporting different platforms.

This structured narrative review summarizes the development history, indications, classification, complications, and recent advances in MCSDs. It emphasizes structured literature selection, guideline-based patient stratification, quantitative outcome data where available, ongoing controversies in the field, and the importance of interpreting domestic clinical experience in the context of international registry benchmarks.

## 2 Methods: structured narrative review approach

This structured narrative review adheres fully to the Standards for Narrative Reviews in Cardiovascular Medicine (SANRA) without quantitative meta-analysis. We searched seven databases: PubMed/MEDLINE, Embase, Web of Science, Cochrane Library, CNKI, Wanfang, and VIP. Two search periods were applied: primary screening covered human clinical studies published 1 January 2000–31 March 2026; snowball reference tracing selectively included pre-2000 landmark MCSDs studies for historical background only, excluding non-pivotal early observational work.

Full reproducible database-specific Boolean search strings, search fields, operators, last search dates, and uniform filters (language, adult population, publication types) are detailed in Supplementary Table S1. Search terms encompassed all MCSDs modalities and clinical contexts. English searches combined MeSH/Emtree terms and free text; Chinese searches used standardized subject headings aligned with the English search framework.

A predefined evidence hierarchy prioritized overlapping guidelines, registries and reviews: RCTs > multinational registries (e.g., STS INTERMACS) > national real-world registries > long-term single-center cohorts > small observational studies > systematic reviews/meta-analyses. Recent consensus guidelines and meta-analyses were prioritized; manufacturer reports served only as supplementary non-peer-reviewed data. Owing to high heterogeneity across MCSDs and context-dependent patient selection, we conducted qualitative comparative analysis rather than pooled meta-analysis, focusing on clinical outcomes, complications and technical innovations.

Two independent reviewers performed two-stage screening and formal quality appraisal, with disagreements resolved by discussion or adjudication from a senior HF/MCSDs cardiologist. Identical eligibility and bias assessment criteria were applied to Chinese and English literature, supplemented by manual searches of domestic core cardiovascular journals and national guidelines. Supplementary Table S1 provides a full itemized SANRA compliance checklist covering protocol design, bias assessment, transparent search documentation, standardized selection procedures and evidence grading.

## 3 The history of MCSDs

Over the past 70 years, MCSDs have experienced remarkable evolution from experimental prototypes to routine clinical tools for advanced HF. The representative milestones throughout its development are summarized in Table 1 (4–9).

**Table 1:** Major milestones in the development of MCSDs over the past 70 years.

Period	Year	Key events & devices	Clinical significance
Foundational era (1950s–1960s)	1953	Gibbon performed the first cardiopulmonary bypass surgery	Laid the technical foundation for mechanical cardiac support
	1957	Kolff and Akutsu implanted a TAH in a canine model	Verified the feasibility of total mechanical cardiac replacement
	1966	Liotta and DeBakey developed the first clinical VAD	Realized clinical application of VAD
	1967	The world's first human heart transplant was completed	Established the concepts of BTT and BTR
Technical breakthrough era 1970s–1990s)	1976	The first implantable VAD was developed	Achieved miniaturization and implantability of assist devices
	1984	NIH successfully implanted an electric pulsed LVAD	Promoted the electrification iteration of LVAD
	-	Berlin Heart EXCOR launched for pediatric patients	Expanded indications to pediatric heart failure
Clinical popularization era (1990s–2000s)	1998	FDA approved HeartMate VE and Novacor LVAD for BTT	Large-scale clinical promotion of LVAD as a transplant bridge
	2002	FDA approved HeartMate XVE as the first DT device	MCSDs became a reliable long-term alternative to heart transplantation
Modern mature era (21st century)	2017	Abbott HeartMate 3 (fully magnetically levitated centrifugal pump) launched	Became the global gold standard for DT
	2022	Domestic fully magnetically levitated LVAD (Shenzhen CoreMed) obtained approval	Improved accessibility to life-saving mechanical circulatory support therapies
Current status	Present	Wide clinical application of MCSDs	Serves as definitive long-term treatment for transplant-ineligible patients; clinical roles keep expanding

MCSDs, Mechanical circulatory support devices; TAH, Total artificial heart; VAD, Ventricular assist device; NIH, National Institutes of Health; LVAD, Left ventricular assist device; FDA, Food and Drug Administration; BTT, Bridge to transplant therapy; BTR, Bridge to recovery; DT, Destination therapy.

## 4 Overview of MCSDs

After over 50 years of technological development, MCSDs have evolved into advanced therapeutic tools that provide partial or complete cardiac replacement for acute and chronic circulatory failure (Table 2) (10, 11).

**Table 2:** Practical comparison of major MCSD categories.

Category	Short-term percutaneous support	Short-term extracorporeal support	Durable support	Biventricular replacement
Typical devices	IABP; Impella; TandemHeart	VA-ECMO; CentriMag temporary surgical VAD	HeartMate II; HeartMate 3; CH-VAD; EVAHEART; CorHeart-type platforms	SynCardia TAH
Usual clinical setting	Acute HF/CS; high-risk PCI; BTD	Profound CS; biventricular failure; postcardiotomy shock; severe cardiorespiratory failure	Advanced chronic HF as BTT/BTD/DT	Selected patients with refractory biventricular failure, often as BTT
Main strengths	Rapid deployment; hemodynamic stabilization; some devices unload the LV	High-flow support; oxygenation with VA-ECMO; flexible escalation	Ambulation; improved perfusion and quality of life; prolonged support duration	Complete replacement of native ventricular function
Key limitations/cautions	Limited durability; vascular complications; bleeding; device-specific support ceiling	Bleeding; stroke; limb ischemia; hemolysis; infection; VA-ECMO increases LV afterload unless unloaded	Pump thrombosis; stroke; GI bleeding; driveline infection; right heart failure; cost and access issues	Extensive surgery; bleeding/infection risk; limited candidate pool; resource intensive
Typical duration of support	Hours to weeks (up to 4–6 weeks)	Days to months (up to 3–6 months for temporary VADs; VA-ECMO typically days to weeks)	Months to years (up to 5–10 + years with modern devices)	Months to years (up to 5 + years in selected patients)
Need for oxygenation	None (isolated hemodynamic support; no integrated oxygenation)	VA-ECMO provides full cardiopulmonary support (integrated oxygenation); temporary surgical VADs provide isolated hemodynamic support (no oxygenation)	None (isolated left ventricular hemodynamic support; no integrated oxygenation)	None (complete biventricular circulatory support; no integrated oxygenation)
LV unloading capacity	Variable; Impella/TandemHeart provide direct LV unloading; IABP provides indirect unloading	Limited with standard VA-ECMO (may increase LV afterload); requires adjunctive unloading (e.g., Impella, atrial septostomy) for optimal LV decompression; temporary VADs provide direct unloading	High; continuous-flow devices provide complete and sustained LV unloading, reducing myocardial wall stress	Complete; TAH replaces native ventricular function, eliminating LV volume and pressure overload
Invasiveness/access route	Minimally invasive; percutaneous femoral/subclavian/axillary arterial access	VA-ECMO: minimally invasive percutaneous femoral/jugular access; temporary surgical VADs: invasive, requires median sternotomy/thoracotomy	Invasive; requires median sternotomy for device implantation; some newer devices offer less invasive surgical approaches	Highly invasive; requires median sternotomy, cardiopulmonary bypass, and native ventricular excision
Typical bridge strategy	BTD; BTC; short-term stabilization for high-risk PCI	BTD; BTC; BTR in postcardiotomy shock	BTT; BTD; long-term DT for ineligible transplant candidates	BTT for patients with refractory biventricular failure ineligible for conventional VAD therapy
Evidence strength	Moderate to high; multiple RCTs and large observational studies support efficacy in CS and high-risk PCI	Moderate; large observational studies support efficacy in refractory CS, limited RCT data for VA-ECMO in CS	High; multiple landmark RCTs and long-term registry data demonstrate improved survival, functional capacity, and quality of life in advanced HF	Moderate; large observational studies and registry data support efficacy in highly selected patients, limited RCT data

IABP, Intra-aortic balloon pump;VA-ECMO, Veno-arterial extracorporeal membrane oxygenation; VAD, Ventricular assist device; CH-VAD, ChinaHeart Ventricular assist device; TAH, Total artificial heart; HF:Heart failure; CS, Cardiogenic shock; PCI, Percutaneous Coronary Intervention; BTD, Bridge to decision; BTT, Bridge to transplant therapy; DT, Destination therapy; LV, left ventricle; GI, Gastrointestinal; BTC, bridge-to-candidacy; BTR, Bridge to recovery; RCT, Randomized Controlled Trial.

## 5 Temporary MCSDs in CS

Temporary MCSDs are mainly indicated for patients with acute HF or CS, particularly those with acute hemodynamic deterioration refractory to pharmacologic therapy. Their principal goals are to restore systemic perfusion, reduce ischemic injury, provide time for revascularization or recovery, and serve as a bridge to decision, durable support, or transplantation. Because the evidence base differs substantially among devices and patient populations, direct superiority claims should be avoided unless supported by adequately matched comparative data or randomized trials.

### 5.1 IABP

IABP is typically inserted via the femoral artery with the balloon catheter placed in the descending thoracic aorta. Its counterpulsation mechanism increases diastolic coronary perfusion and decreases systolic left ventricular afterload, featuring rapid bedside deployment, wide availability, and acceptable safety. Nevertheless, IABP provides only modest hemodynamic support. The landmark IABP-SHOCK II trial confirmed that routine IABP use does not improve 30-day or long-term survival in patients with severe acute myocardial infarction (AMI)-related CS (12). In line with this pivotal evidence, 2025 American College of Cardiology/American Heart Association (ACC/AHA) and 2023 European Society of Cardiology (ESC) ACS guidelines provide Class III recommendation against routine IABP in AMI-CS, indicating no net clinical benefit (13).

Therefore, modern clinical practice limits IABP use to highly selective, adjunctive, or bridging MCSDs scenarios. Valid selective indications include serving as a surgical bridge for AMI-related mechanical complications (ventricular septal defect, papillary muscle rupture), rescuing persistent myocardial ischemia after successful PCI, stabilizing high-risk pre-shock or early CS (SCAI A/B stage), and acting as a temporary alternative when advanced MCSDs are unavailable or unsuitable (14).

Major contraindications of IABP consist of severe aortic insufficiency (AI) and advanced peripheral arterial disease (PAD). Common procedural complications include access-site bleeding, limb ischemia, stroke, and local or systemic infection.

### 5.2 ECMO

ECMO provides temporary cardiopulmonary support by draining systemic venous blood, performing extracorporeal oxygenation, and recirculating purified blood into the systemic circulation (Expert consensus/guideline recommendations) (15, 16). The two major ECMO modalities exhibit distinct clinical indications: veno-venous ECMO (VV-ECMO) is solely used for isolated refractory respiratory failure without circulatory impairment and is not discussed in this work focusing on heart failure and circulatory support. By contrast, VA-ECMO acts as a core mechanical circulatory support tool, which is widely applied for refractory CS, postcardiotomy shock, fulminant myocarditis, and selected cases of refractory cardiac arrest or BTT.

VA-ECMO delivers simultaneous high-flow systemic perfusion and complete gas exchange for rescue therapy in reversible refractory CS. However, patients receiving VA-ECMO present with critical baseline illness and extensive comorbidities, complicating outcome assessment. Broad claims of universal survival benefit require etiology-specific refinement. The pivotal ECLS-SHOCK trial confirms that routine early VA-ECMO fails to lower mortality in AMI-related CS and may raise severe adverse event risks (17). Survival advantages are limited to specific reversible shock phenotypes, necessitating individualized risk stratification instead of blind empirical deployment.

VA-ECMO's risk and efficacy vary substantially across six clinical subgroups: (1) AMI-related CS (no survival benefit with routine early implantation); (2) fulminant myocarditis (high rates of BTR); (3) transient postcardiotomy shock; (4) decompensated chronic HF with advanced end-organ injury; (5) isolated biventricular failure without severe hypoxemia; (6) combined cardiorespiratory collapse requiring dual circulatory and respiratory support.

Common VA-ECMO adverse events encompass major bleeding, stroke, acute kidney injury, limb ischemia, hemolysis and secondary infection. Peripheral VA-ECMO inherently insufficiently unloads the LV: retrograde aortic flow increases LV afterload, aggravates LV dilatation and dysfunction, and worsens pulmonary edema. Adjunct LV unloading strategies (IABP, Impella, surgical ventricular venting, atrial septostomy) alleviate this hemodynamic burden, though the optimal combined regimen remains controversial amid evolving consensus evidence (Expert consensus/guideline recommendations) (18). Importantly, observational comparisons between Impella and VA-ECMO are heavily confounded by disparities in illness severity, ventricular morphology, respiratory compromise, implantation timing and therapeutic goals, restricting reliable causal conclusions from registry data alone.

Peripheral arterial cannulation may trigger distal limb hypoperfusion, ischemia and necrosis. Continuous distal perfusion surveillance is required, and prophylactic distal perfusion catheters are indicated for high-risk patients to avoid limb loss. Given etiology-dependent variations in mortality and complication risks, VA-ECMO implantation requires thorough phenotype-tailored risk–benefit evaluation.

### 5.3 Impella

Impella is a percutaneous or surgical microaxial MCSD that crosses the aortic valve to directly unload the LV and boost systemic cardiac output (19). Unlike IABP's passive cycle-synchronized counterpulsation, Impella delivers sustained active circulatory support unaffected by tachyarrhythmias or electromechanical dissociation, independent of ECG or arterial waveform triggers.

It serves two core indications: hemodynamic backup for high-risk PCI and short-term rescue of STEMI-related CS requiring urgent LV decompression. Impella efficacy data are highly population-specific and cannot be extrapolated to all shock phenotypes. The DanGer Shock RCT confirmed reduced 6-month all-cause mortality with early Impella CP only in isolated STEMI CS without severe right ventricular dysfunction or refractory hypoxemia (20). This survival benefit comes with substantially higher risks of major bleeding, limb ischemia, renal replacement therapy, hemolysis and sepsis. Such trial results do not apply to patients with dominant RV failure, persistent refractory shock, MI mechanical complications, severe hypoxemia, fulminant myocarditis, or postcardiotomy shock.

Propensity-matched and observational studies show better short-term survival and fewer device complications with Impella vs. VA-ECMO in select CS patients, yet these comparisons carry marked selection bias: VA-ECMO is reserved for those with advanced biventricular failure or critical hypoxemia (21). Accordingly, contemporary MCSDs selection follows phenotype-guided individualized strategies instead of universal device superiority.

Impella-associated adverse events encompass access-site bleeding, device malposition, aortic valve damage, arrhythmia, pump thrombosis, hemolysis, stroke and peripheral limb ischemia. Key uncertainties persist about optimal implantation timing, uniform inclusion criteria and standardized patient stratification. Large prospective randomized trials across heterogeneous shock subgroups are needed to optimize personalized Impella use and validate long-term risk–benefit profiles.

### 5.4 TandemHeart device

TandemHeart is a percutaneous transseptal VAD that shunts oxygenated left atrial blood into the systemic arterial circulation, delivering superior LV unloading and circulatory support compared with IABP (22, 23). It is primarily indicated for severe acute HF, CS, high-risk PCI, and temporary mechanical bridging before definitive cardiac intervention. Despite its favorable hemodynamic performance, the clinical adoption of TandemHeart remains limited worldwide, which is largely attributed to inherent procedural and clinical drawbacks as well as center-dependent technical requirements (24, 25). Its implantation necessitates sophisticated transseptal puncture, a technically challenging procedure with a steep learning curve that is considerably more complex than IABP and Impella placement (23, 25). Moreover, the device carries a distinct risk profile involving bleeding, limb ischemia, thromboembolism, hemolysis, and persistent atrial septal defects (ASD), further restricting its widespread use. Competent implantation and postoperative management also rely heavily on specialized institutional experience and proficient operator expertise, confining its application to high-volume specialized cardiac centers (24).

To date, clinical evidence supporting TandemHeart application is mainly obtained from observational registries, while high-quality RCTs remain scarce (22, 24). Overall, TandemHeart achieves superior LV unloading at the cost of higher procedural complexity and complication risks relative to competing mechanical support devices. Several key clinical questions also remain unanswered, including the optimal timing of device initiation, appropriate support duration, and long-term outcomes of residual ASD. Combined analysis of real-world registry data and rigorous randomized trial evidence is therefore essential to standardize its clinical application and refine patient selection strategies.

### 5.5 CentriMag device

CentriMag system serves as a temporary MCSD designed for short-term surgical intervention. Its primary role is to provide immediate hemodynamic stabilization in critically ill patients who cannot be weaned from cardiopulmonary bypass. To date, there are no published RCTs of CentriMag. The highest-quality available evidence is derived from prospective single-arm pivotal trials and large multicenter observational cohorts, categorized as Level B-NR by ACC/AHA criteria. In postcardiotomy shock, CentriMag offers temporary left, right, or biventricular support when the heart fails to recover adequate function following cardiac surgery (26). For isolated right ventricular (RV) failure, the device can be configured to provide dedicated RV support, addressing the specific hemodynamic derangements associated with right-sided heart dysfunction (27). When both ventricles require assistance, biventricular support can be achieved through dual-pump configurations, maintaining systemic and pulmonary circulation simultaneously (26). Additionally, CentriMag functions as a BTD modality, stabilizing patients while the clinical team evaluates options for recovery, transition to long-term VAD therapy, or candidacy for cardiac transplantation (28).

Multiple ongoing controversies remain central to the clinical application of temporary MCSDs across different shock etiologies. A key debate lies in the timing of MCSDs initiation for CS, specifically whether early intervention or delayed therapeutic escalation yields superior clinical outcomes (20). Additionally, the value of dedicated shock response teams in standardizing care and improving patient prognosis continues to be discussed in clinical practice (29). For VA-ECMO, the necessity of concomitant LV unloading remains a contentious topic, with inconsistent evidence regarding its net clinical benefit (30). Clinicians also face a persistent trade-off between adequate hemodynamic support and the elevated risk of device-related complications, which complicates clinical decision-making (16). Furthermore, optimal temporary MCSD selection varies distinctly based on the underlying cause of CS, including AMI, acute myocarditis, postcardiotomy shock, and decompensated chronic HF (18). Notably, direct head-to-head comparisons between Impella and VA-ECMO should be interpreted with extreme caution, as the patient populations receiving these two devices differ substantially in baseline characteristics, creating inherent limitations for such comparative analyses (Table 3) (21).

**Table 3:** Summary of pivotal RCTs evaluating temporary MCSDs in CS.

	IABP-SHOCK II	ECLS-SHOCK	DanGer Shock
Study population	AMI complicated by CS	Infarct-related CS(with or without refractory status/cardiac arrest)	STEMI with refractory CS
Device	IABP vs. no IABP	VA-ECMO plus standard therapy vs. standard therapy alone	Impella CP plus guideline-directed therapy vs. guideline-directed therapy alone
Primary endpoint	30-day all-cause mortality	30-day all-cause mortality	180-day all-cause mortality
Major efficacy result	No significant difference (39.7% vs. 41.3%, *P* = 0.69)	No significant difference (47.8% vs. 49.0%, RR 0.98, *P* = 0.81)	Significant mortality reduction (45.8% vs. 58.5%, HR 0.74, *P* = 0.04)
Key safety findings	No significant differences in major bleeding, stroke, or sepsis	Higher moderate/severe bleeding (23.4% vs. 9.6%, RR 2.44) and peripheral vascular complications (11.0% vs. 3.8%, RR 2.86)	Higher composite adverse safety events (24.0% vs. 6.2%, RR 4.74); more bleeding and renal replacement therapy requirement
Principal limitations	MI-related CS only; excludes patients requiring high-level circulatory support	Small sample size; protocol-permitted crossover; heterogeneous shock phenotypes	Limited to refractory STEMI-CS; low generalizability to non-ischemic CS

RCTs, Randomized controlled trials; MCSDs, Mechanical circulatory support devices; CS, Cardiogenic shock; AMI, Acute myocardial infarction; STEMI, ST-elevation myocardial infarction; IABP, Intra-aortic balloon pump; VA-ECMO, Veno-arterial extracorporeal membrane oxygenation.

## 6 Durable MCSD in advanced HF

Durable MCSD generally refers to devices providing support for months to years, predominantly durable LVADs, with TAH reserved for selected patients with refractory biventricular failure. These platforms have transformed care for advanced HF by enabling ambulation, end-organ recovery, and prolonged survival in appropriately selected patients. Nevertheless, durable MCSDs remains a high-risk, high-cost therapy that requires careful candidate selection and lifelong surveillance. Contemporary decision-making must weigh expected survival benefit and quality-adjusted life years against recurrent hospitalization, adverse events, caregiver burden, and health-system affordability (31).

Indications for durable MCSD include inotrope dependence, refractory congestion or hypoperfusion despite guideline-directed medical and device therapy, recurrent HF hospitalizations, poor exercise tolerance, progressive cachexia, and potentially reversible end-organ dysfunction due to low output (18). In contrast, severe irreversible liver or kidney failure, advanced frailty, uncontrolled infection, major psychosocial barriers, or inability to adhere to anticoagulation and driveline care may indicate prohibitive risk or futility. These considerations are as important as technical operability.

### 6.1 HeartMate II (HM II) device

As a second-generation continuous-flow axial pump, HeartMate II (HM II) marked a pivotal evolution from first-generation pulsatile LVADs, featuring a more compact profile and substantially enhanced mechanical durability. It quickly became a mainstream choice for both BTT and DT, cementing durable LVAD as a standard therapeutic strategy for patients with end-stage HF. Equipped with ruby bearings for heat dissipation and titanium microbead coatings on blood-contacting surfaces and tubing connectors, HM II improves hemocompatibility and minimizes blood cell injury. The device obtained U.S. FDA approval for BTT in 2008 and DT in 2010, and has been implanted in over 26,600 patients worldwide to date (32).

Real-world registry data from the International Society for Mechanical Circulatory Support (ISMCS) demonstrated an 80% 1-year survival rate and a 69% 2-year survival rate among HM II recipients (33). Despite its clinical success, HM II is hampered by inherent limitations linked to its axial-flow design and bearing system. The most prominent adverse event is pump thrombosis, with its incidence rising from 2.2% to 8.4% within the first three months post-implantation (34). Concomitant complications including ischemic/hemorrhagic stroke, GI bleeding and driveline infection also remain prevalent. While optimized anticoagulation regimens (target international normalized ratio 2.0–3.0) and perioperative heparin bridging can partially mitigate the risks of pump thrombosis and stroke, these issues could not be fully resolved with second-generation technology. Collectively, the unmet clinical needs of axial-flow pumps drove the paradigm shift toward third-generation fully magnetically levitated centrifugal LVADs.

### 6.2 HeartMate 3 (HM3) device

HeartMate 3 (HM3) represents the third generation of durable LVAD, adopting a fully magnetically levitated centrifugal design that eliminates mechanical bearings entirely. This fundamental technical upgrade distinguishes it from the axial-flow HM II and delivers tangible improvements in clinical safety and long-term outcomes (35). Multiple high-quality clinical studies and large registries have validated the advantages of HM3 across key efficacy and safety endpoints.

The landmark MOMENTUM 3 trial established the clinical superiority of HM3 relative to HM II at the 2-year follow-up, and extended 5-year data further confirmed sustained clinical benefits (36). Compared with HM II, HM3 achieves remarkable reductions in three major adverse events: device thrombosis is nearly eliminated, the incidence of disabling stroke is significantly lowered, and the requirement for pump-related reoperation is markedly decreased (Table 4). Data from the Society of Thoracic Surgeons (STS) INTERMACS registry also corroborated that magnetically levitated centrifugal pumps are associated with superior short-term and intermediate-term survival, as well as an overall reduced adverse event burden vs. earlier axial-flow platforms (37).

**Table 4:** Quantitative comparison of clinical outcomes between HeartMate II and HeartMate 3 at 1-year and 2-year follow-up (data from MOmenTUM 3 RCT).

Outcome endpoint	Follow-up	HeartMate II (second-generation axial-flow pump)	HeartMate 3 (third-generation magnetically levitated centrifugal pump)
Overall survival	1 year	80.0%	85.1%
Overall survival	2 years	69.3%	79.5%
Pump thrombosis	1 year	8.4%	0.8%
Disabling stroke	2 years	12.1%	5.0%
Pump-related reoperation	2 years	14.2%	3.5%
Major bleeding	1 year	39.6%	37.2%
GI bleeding	1 year	18.7%	21.4%
Driveline infection	2 years	17.5%	16.8%
RV failure	1 year	14.3%	13.9%
All-cause hospital readmission	1 year	62.8%	60.5%

RCT, Randomized controlled trial; RV, Right ventricular; GI, Gastrointestinal.

Notably, although the advanced design of HM3 has greatly improved device-related safety and survival profiles, several persistent complications still challenge long-term durable LVAD support. GI bleeding, systemic and driveline infection, RV failure and unplanned hospital readmissions continue to occur at considerable rates. These observations indicate that while technological innovation has alleviated many drawbacks of traditional LVADs, it cannot completely resolve the inherent trade-offs associated with prolonged MCSDs.

Consistent with the quantitative data summarized in Table 3, HeartMate 3 exhibits superior hemocompatibility compared with HeartMate II, reflected by substantially lower 1- and 2-year rates of pump thrombosis and pump-associated reoperation. Nevertheless, this clinical advantage does not eliminate major adverse complications. GI bleeding, driveline infection, right heart failure and recurrent hospital readmission remain common adverse events in patients implanted with HeartMate 3, with event rates comparable to the HeartMate II cohort at matched follow-up timepoints. These persistent complications impose substantial long-term clinical and economic burdens and should not be downplayed when evaluating the overall clinical performance of magnetically levitated centrifugal flow pumps. Future real-world registry studies are required to further characterize the long-term incidence and risk factors of these unresolved adverse events.

### 6.3 Total artificial heart (TAH) device

The SynCardia TAH is the only clinically approved implantable MCSD that enables complete biventricular functional replacement. It is indicated exclusively for carefully selected patients with end-stage, irreversible biventricular HF who fail maximal medical therapy and conventional surgical intervention (38). Current clinical evidence for TAH is restricted to observational registry data and single-center cohort studies, and no prospective RCTs have validated its comparative efficacy or refined patient selection criteria (39). For patients with refractory biventricular failure, registry data has shown satisfactory BTT outcomes, with a 79% BTT success rate and a 70% one-year post-transplant survival rate.

Despite its satisfactory BTT efficacy, TAH therapy has notable limitations and imposes substantial burdens on healthcare resources, primarily due to prolonged hospitalization. Its major severe complications involve perioperative bleeding, device or systemic infection, ischemic stroke, renal dysfunction, thrombosis, and chronic anemia. Among these, postoperative infection serves as a critical modifiable factor affecting long-term patient survival. Different from LVADs, which only ameliorate isolated left-sided HF, TAH restores complete biventricular circulatory function (40). However, its high complication risks and rigorous clinical management requirements limit its broader clinical application compared with less invasive alternative MCSD strategies.

TAH implantation is limited to highly screened patients presenting with severe biventricular failure, irreversible ventricular arrhythmias, or anatomical and physiological conditions that preclude standalone LVAD implantation (41). Typical eligible clinical scenarios cover severe myocardial infarction complicated by septal rupture, refractory angina with inoperable coronary lesions, and congenital heart defects incompatible with single-ventricle device implantation. Unlike LVADs, which are widely used for destination therapy, TAH implantation requires rigorous multidisciplinary evaluation to confirm that biventricular replacement is the only viable therapeutic option for individual patients.

### 6.4 Regional/domestic platforms

#### 6.4.1 CH-VAD

The CH-VAD, a domestically developed third-generation fully magnetically levitated centrifugal LVAD, obtained National Medical Products Administration (NMPA) marketing approval in 2021. Its clinical evidence originates from two separate datasets: a 2017 single-center feasibility pilot and a later multicenter real-world cohort (42, 43).

The 2017 pilot enrolled just four young adults (23–46 years) with follow-up shorter than 200 days. It showed promising short-term hemodynamics, compact design and acceptable early safety. Two patients with reversible dilated cardiomyopathy achieved successful device explantation after 116 and 193 days of support, while one patient with acute myocarditis died from non-device-related causes, precluding definitive conclusions for myocarditis-related HF. No device malfunction, pump thrombosis, stroke or major bleeding occurred in this small cohort, yet results are severely limited by its tiny sample size and brief surveillance.

The multicenter real-world cohort includes a larger population of patients with end-stage HF and longer median follow-up, reporting standardized 6-month, 1-year and 2-year survival, plus quantified rates of adverse events: stroke, surgical/GI bleeding, pump thrombosis, driveline infection, *de novo* RV failure, unplanned HF rehospitalization and device malfunction (43). Relative to international fully magnetically levitated LVAD registries (e.g., HeartMate series), short-term survival and bleeding risks are numerically similar, whereas late pump thrombosis and long-term right HF rates show mild discrepancies. No prospective head-to-head randomized trials comparing CH-VAD with HeartMate devices, VA-ECMO or IABP have been published to date.

Major evidence gaps remain despite the multicenter dataset. First, the aggregate patient population across all CH-VAD studies is far smaller than global pivotal LVAD trials and long-term registries. Second, inconsistent definitions of long-term adverse outcomes across Chinese cohorts limit cross-study pooled analysis. Third, data on device durability beyond 2 years, long-term complication trajectories, and performance across diverse HF etiologies (ischemic cardiomyopathy, chronic myocarditis, etc.) are insufficient. Furthermore, the single-center pilot cannot be extrapolated to general end-stage HF populations given its narrow inclusion of young patients with reversible cardiomyopathy.

Without multinational registries or randomized comparative trials, current single-center pilot and multicenter observational CH-VAD data require cautious interpretation. Large-scale long-term multicenter registries with unified adverse event definitions, prolonged follow-up and matched cohorts against international LVAD benchmarks are needed to validate CH-VAD's long-term efficacy, durability and real-world generalizability.

#### 6.4.2 EVAHEART series

The EVAHEART series are key domestically optimized MCSDs in China, reducing dependence on imported LVADs like the HeartMate platform. The original EVAHEART prototype was developed in Japan; EVAHEART I and EVAHEART 2 discussed herein are independently produced, China-specific variants with separate NMPA approval pathways. All cited clinical data derive solely from Chinese patients, and findings from the Japanese prototype cannot be extrapolated to these domestic iterations.

As China's first domestically commercialized artificial heart, the first-generation EVAHEART I obtained clinical trial approval in August 2017 and full marketing clearance in August 2019. Weighing 420 g, this pure-titanium pulsatile centrifugal LVAD features a 40 mm impeller rotating at 1,800–2,400 rpm with a maximal flow of 10–15 L/min. Its proprietary water-sealing system combines bearing lubrication and heat dissipation to produce native cardiac-synchronized pulsatile flow, differing fundamentally from continuous-flow LVADs.

Regulatory clearance and early surgical feasibility do not confirm comparative efficacy against imported LVADs. All EVAHEART I clinical data come from small single-center single-arm registries with brief follow-up, without multicenter cohorts or prospective RCTs. Current evidence only supports short-term perioperative safety, while long-term survival and standardized adverse event profiles remain poorly defined.

Chinese single-center registries report highly variable rates of stroke, pump thrombosis, bleeding, device infection and unplanned rehospitalization, absent standardized international comparative benchmarks (44). A major design flaw of EVAHEART I is its transventricular drainage cannula, creating a thrombogenic surface that increases risks of pump thrombosis and ischemic stroke in domestic patients (44).

The revised EVAHEART 2 adopts a non-penetrating dual suture-ring cannula, substantially lowering thrombosis, stroke and ventricular septal deviation vs. EVAHEART I. Still, long-term real-world hazards (device infection, GI bleeding, readmission) and long-term economic costs lack robust quantitative data from Chinese cohorts, with no cross-reference to Japanese prototype results.

These knowledge gaps limit widespread standardized use of EVAHEART I. Single-center registries suffer from selection bias and lack control arms, failing to prove non-inferiority or superiority over imported MCSDs. Large multicenter RCTs and long-term national registries are necessary to verify the generalizability, long-term safety, cost-effectiveness and unique clinical value of China's localized EVAHEART series, separate from early Japanese prototype data.

## 7 Device selection and clinical decision-making

Clinically, MCSD application follows a staged framework based on support duration and therapeutic intent. Temporary MCSDs are mainly used for acute HF and CS, whereas durable MCSDs are indicated for carefully selected patients with end-stage chronic HF as BTT, BTC/BTD, BTR, or DT. Contemporary guidelines emphasize scenario-specific implantation strategies, adopting the Society for Cardiovascular Angiography and Interventions (SCAI) shock staging for acute CS and the Interagency Registry for Mechanically Assisted Circulatory Support (INTERMACS) profiling for advanced chronic HF, as disease severity and clinical timing substantially affect patient outcomes (Tables 5, 6) (10, 11).

**Table 5:** Expert-synthesized clinical decision framework for phenotypic stratification of CS (acute CS/SCAI stages).

Clinical Stratification	Core Clinical Phenotype	Key Clinical Decision Context for MCSDs Selection	Evidence-Informed Clinical Framework Considerations	Supporting Guideline Reference
SCAI Stage A (At-risk CS)	Patients at risk of CS without current evidence of shock; HF with hypotension/rising cardiac biomarkers, no overt end-organ hypoperfusion, in a pre-shock state at risk of imminent deterioration	Shock etiology and risk of progression Baseline ventricular function Presence of acute decompensated HF triggers Patient's treatment goals	No urgent MCSDs indicated; priority is medical optimization and close hemodynamic monitoring. Standby temporary percutaneous MCSDs may be considered for imminent deterioration, based on individual risk stratification	2022 SCAI Expert Consensus
SCAI Stage B (Beginning CS)	Beginning CS with hemodynamic instability, early evidence of shock, without clear overt hypoperfusion; may have mild renal/hepatic injury, responsive to initial vasopressor/inotrope therapy	Shock etiology (ischemic vs. non-ischemic) Hemodynamic trajectory and stability Response to initial pharmacotherapy Need for ongoing monitoring vs escalation	MCSDs are not routinely first-line; priority is optimization of pharmacotherapy and hemodynamic monitoring. For patients with progressive instability despite maximal pharmacotherapy, escalation to temporary percutaneous MCSDs may be considered, with modality selection tailored to individual clinical context	2022 SCAI Expert Consensus
SCAI Stage C (Classic CS)	Persistent CS with end-organ hypoperfusion requiring high-dose vasopressors/inotropes; refractory to standard pharmacotherapy	Left vs right ventricular predominant failure Need for oxygenation support Degree of LV unloading required Vascular access suitability Shock reversibility	Temporary percutaneous MCSDs may be considered for hemodynamic support, with modality selection individualized based on ventricular phenotype, end-organ function, and treatment goals. Upfront durable LVAD implantation is not recommended in the acute shock phase	2022 SCAI Expert Consensus
SCAI Stage D (Deteriorating CS)	Progressive CS with worsening multiorgan dysfunction despite initial MCSDs support; refractory hemodynamic collapse	Biventricular failure vs isolated LV failure Need for concurrent oxygenation support Degree of LV distension and unloading requirement Neurological prognosis Potential for recovery or bridge to long-term support	Combination MCSDs strategies may be considered for refractory hemodynamic collapse, with selection tailored to the patient's ventricular phenotype, end-organ function, and treatment goals. LV unloading should be considered in patients with VA-ECMO to prevent LV distension	2022 SCAI Expert Consensus
SCAI Stage E (Refractory CS)	CS with refractory hemodynamic collapse, progressive multiorgan dysfunction; clinical status may be reversible with aggressive rescue therapy in appropriate clinical contexts	Neurological prognosis Shock etiology and potential reversibility Patient's pre-morbid status and treatment goals Availability of long-term support options	Emergency MCSDs may be considered as a rescue therapy for selected patients with potentially reversible shock, with decisions individualized based on neurological prognosis, treatment goals, and potential for recovery or bridge to long-term support. MCSDs is not indicated for patients with irreversible neurological injury or no potential for meaningful recovery	2022 SCAI Expert Consensus

CS, Cardiogenic shock; HF, Heart failure; MCSDs, Mechanical circulatory support devices; SCAI, Society for Cardiovascular Angiography and Interventions; VA-ECMO, Veno-arterial extracorporeal membrane oxygenation; LV, Left ventricle; LVAD, Left ventricular assist device.

**Table 6:** Expert-synthesized clinical decision framework for phenotypic stratification of CS (chronic HF/INTERMACS profiles) .

Clinical Stratification	Core Clinical Phenotype	Key Clinical Decision Context for MCSDs Selection	Evidence-Informed Clinical Framework Considerations	Supporting Guideline Reference
INTERMACS 1 (Crashing CS)	Chronic HF with acute refractory shock, dependent on inotropes/emergency MCSDs; critical hemodynamic instability	Shock etiology and reversibility Candidate status for transplant/durable LVAD End-organ function Treatment goals	Temporary MCSDs (VA-ECMO/Impella) may be considered as a BTR or BTT evaluation, with modality selection tailored to individual clinical context	2023 ISHLT MCSDs Guideline
INTERMACS 2 (Progressive Decline)	Dependent on high-dose chronic inotropes, recurrent unstable HF, progressive end-organ dysfunction	Durable MCSDs candidacy Right ventricular function Vascular access suitability Patient's treatment goals	Temporary percutaneous MCSDs may be considered as a bridge to durable LVAD implantation, with priority given to early evaluation for long-term MCSDs in eligible patients	2023 ISHLT MCSDs Guideline
INTERMACS 3 (Stable on Inotropes)	Continuous low-dose inotrope dependence, recurrent HF hospitalization, no acute shock	Durable MCSDs candidacy Right ventricular function Patient's functional status and treatment goals	Primary durable LVAD implantation (BTT/DT) may be considered as first-line chronic MCSDs in eligible patients, with optimal GDMT continued as adjunctive therapy	2023 ISHLT MCSDs Guideline
INTERMACS 4–7 (Ambulatory Advanced HF)	Non-inotrope dependent chronic HF with varying exercise limitation; no acute shock or end-organ hypoperfusion	HF progression trajectory Functional status Durable MCSDs candidacy Patient's treatment goals	Optimal GDMT is first-line therapy. Elective durable LVAD implantation may be considered only for eligible patients with progressive deterioration despite maximal medical therapy	2023 ISHLT MCSDs Guideline

CS, Cardiogenic shock; HF, Heart failure; MCSDs, Mechanical circulatory support devices; VA-ECMO, Veno-arterial extracorporeal membrane oxygenation; LV, Left ventricle; LVAD, Left ventricular assist device; INTERMACS, Interagency Registry for Mechanically Assisted Circulatory Support; ISHLT, International Society for Heart and Lung Transplantation; BTR, Bridge to recovery; BTT, Bridge to transplant therapy; DT, Destination therapy; GDMT, Guideline-Directed Medical Therapy.

These two tables represent expert-synthesized clinical decision framework for phenotypic stratification of CS, not a mandatory guideline-based device selection algorithm. MCSDs selection must be individualized based on shock etiology, ventricular phenotype, need for oxygenation, degree of LV unloading required, vascular access suitability, neurological prognosis, shock reversibility, and intended bridge strategy (BTR/BTT). No specific MCSDs modality is routinely indicated solely on the basis of SCAI or INTERMACS staging alone.

Optimal MCSDs selection relies on multidisciplinary evaluation by cardiologists, intensive care clinicians, cardiac surgeons, perfusionists, and specialized nurses. Key decision-making factors encompass shock severity, anticipated support duration, oxygenation demand, ventricular unloading needs, vascular accessibility, bleeding and thrombotic risks, neurological function, frailty, end-organ dysfunction reversibility, and the likelihood of transplantation, cardiac recovery, or long-term supportive benefit. For acute CS, SCAI staging, lactate levels, vasopressor requirement, and progressive hypoperfusion guide early intervention before refractory shock develops. For advanced chronic HF, INTERMACS profiles, recurrent hospitalization, inotrope dependence, impaired exercise capacity, malnutrition, and progressive hepatorenal dysfunction determine the timing of durable MCSD implantation. Notably, irreversible end-organ injury, severe neurological impairment, uncontrolled infection, and major socioeconomic barriers preclude beneficial MCSD implantation, despite technical operability.

## 8 Complications and long-term trade-offs

MCSDs serve as a pivotal therapeutic strategy for end-stage HF, enabling the correction of acute hemodynamic collapse, restoration of systemic organ perfusion, and provision of bridging support for patient recovery, clinical decision-making, heart transplantation, or long-term durable mechanical therapy. Despite these definitive clinical benefits, MCSD implantation and long-term support are associated with a high burden of adverse events, which substantially compromise patient prognosis and quality of life. Notably, the incidence, timing, spectrum, and pathogenic characteristics of MCSD-related complications vary significantly by device classification and support duration. Temporary MCSDs are primarily applied for short-term hemodynamic rescue, whereas durable LVADs are designed for long-term chronic support, with distinct complication profiles between the two device categories. In general, early perioperative complications (within 30 days post-implantation) are dominated by bleeding, vascular injury, severe hemolysis, and acute infection, while late long-term complications (beyond 30 days and during chronic support) predominantly include thromboembolic events, stroke, device thrombosis, GI bleeding, AI, driveline-related infection, and progressive device malfunction. This section systematically categorizes and elaborates on MCSD complications by device type and disease course, with a focus on device-specific pathogenesis, clinical characteristics, and management strategies.

### 8.1 Early perioperative complications (temporary and durable MCSDs)

Early complications occur in the acute perioperative stage after all MCSD implantations, regardless of device type, and are closely associated with surgical trauma, cardiopulmonary bypass stimulation, acute coagulation disorders, and intraoperative vascular injury. These acute adverse events are the leading causes of early postoperative mortality and prolonged intensive care unit (ICU) stay in MCSD recipients.

#### 8.1.1 Hemorrhage

Bleeding is the most common early complication after MCSD implantation, attributed to surgical trauma, perioperative anticoagulation, and end-organ dysfunction-induced acquired coagulopathy. For patients with durable continuous-flow LVADs, bleeding risk persists chronically, presenting as recurrent epistaxis, GI bleeding, and life-threatening intracranial hemorrhage.

The management of MCSD-related bleeding requires individualized antithrombotic adjustment, coagulopathy correction, and targeted transfusion, while balancing the competing risks of hemorrhage and device thrombosis. Intracranial hemorrhage combined with cerebral thromboembolism and vascular necrosis creates a critical therapeutic dilemma, as anticoagulation is required for thrombosis prevention whereas adequate coagulation function is essential for hemostasis. For acute postoperative bleeding, anticoagulation should be temporarily withheld and gradually resumed based on patient recovery, with red blood cell transfusion applied for severe hemorrhagic anemia (45, 46).

#### 8.1.2 Acute right heart dysfunction

Acute RV failure is a crucial early complication post-LVAD implantation, associated with prolonged ICU stay, renal dysfunction, hepatic congestion, and poor long-term survival. Key risk factors include preexisting RV dysfunction, elevated pulmonary vascular resistance (PVR), severe tricuspid regurgitation, advanced INTERMACS classification, and perioperative bleeding or massive transfusion. Postoperative acute RV dysfunction is primarily driven by abrupt PVR elevation. Chronic HF induces baseline PVR elevation, and cardiopulmonary bypass further aggravates pulmonary endothelial dysfunction postoperatively. The increased PVR impairs RV filling and systolic function, limiting right-to-left cardiac output delivery and ultimately causing right heart decompensation.

Pulmonary hypertension, a typical complication of chronic HF, further exacerbates postoperative RV insufficiency (47). Its pathogenesis involves chronic left atrial hypertension, pulmonary venous congestion, volume overload, and sympathetic dysregulation (48). Mechanistically, elevated PVR is linked to impaired endothelial nitric oxide release, and cardiopulmonary bypass-related endothelial injury further increases perioperative PVR (49). In LVAD recipients with advanced HF, preexisting pulmonary hypertension restricts device filling, which can be worsened by hemorrhage-induced inflammatory cytokines, often necessitating biventricular support. Elevated central venous pressure from RV dysfunction also increases venous bleeding risk, forming a detrimental hemodynamic cycle (50).

Clinical interventions include pulmonary vasodilator administration, preload/afterload optimization, temporary RV mechanical support, and rigorous preoperative risk stratification. Irreversible severe RV dysfunction abolishes the benefits of durable LVADs and serves as a critical indicator for treatment futility assessment.

#### 8.1.3 Acute vascular injury and hemolysis

Acute vascular injury is a procedure-related complication, predominantly occurring in temporary MCSD patients receiving large-bore cannulation, resulting from intraoperative vascular puncture and mechanical compression. Early postoperative hemolysis arises from mechanical erythrocyte destruction by high-speed pump rotation and abnormal transvalvular gradients, characterized by elevated free hemoglobin and reduced haptoglobin, which aggravates renal injury and coagulation disorders.

### 8.2 Late long-term complications (durable LVAD-specific)

Late complications specifically occur in patients receiving long-term durable LVAD support, typically emerging 30 days after implantation and persisting throughout the chronic support period. These device-specific adverse events are the primary factors limiting long-term patient survival and quality of life, with distinct characteristics different from temporary MCSD-related complications.

#### 8.2.1 Aortic regurgitation

Progressive aortic regurgitation (AR) is a unique chronic complication of continuous-flow LVADs. Persistent transvalvular pressure gradients and reduced native valve opening induce aortic cusp fusion and valvular incompetence, creating a recirculatory loop that reduces effective forward flow and attenuates LVAD hemodynamic benefits. Management strategies include blood pressure control, pump speed optimization, concomitant surgical correction during primary implantation for high-risk patients, and transcatheter intervention for advanced AR.

#### 8.2.2 Device-related infections

Infection remains a major limitation of MCSDs therapy, with distinct profiles across device types (18). Temporary MCSDs are mainly complicated by cannula-site infection and secondary bloodstream infection, which resolve after device removal (51). In contrast, durable LVADs are prone to chronic recurrent infections, including driveline exit-site infection, pump pocket infection, and refractory device-related sepsis.

Prolonged LVAD support, repeated hospitalization, diabetes, malnutrition, and substandard exit-site care are major infection risk factors. Preventive measures involve refined surgical techniques, standardized driveline management, and multidisciplinary long-term follow-up. The porous coating of LVAD cannulas and drivelines facilitates tissue ingrowth and barrier reconstruction at skin exit sites, effectively reducing chronic bacterial infection risk.

#### 8.2.3 Pump thrombosis, thromboembolic stroke and device malfunction

Pump thrombosis and thromboembolic stroke are the most severe late complications of durable LVAD support (18, 52). The upgrade from axial-flow to fully magnetically levitated centrifugal devices has markedly reduced pump thrombosis and reoperation rates, though thromboembolic stroke risk persists and remains a major threat to long-term patient safety.

Thrombotic and thromboembolic events are attributed to multiple factors, including device design, suboptimal anticoagulation, infection, arrhythmia, unstable blood pressure, and poor patient adherence. Although device malfunction, system alarm failure, cable/controller dysfunction, and outflow graft lesions are less common in modern devices, these events still trigger emergent hospitalization and urgent pump exchange.

#### 8.2.4 GI bleeding and quality of life trade-offs

GI bleeding represents a common and unique chronic complication associated with continuous-flow LVAD support, which is primarily attributed to long-term anticoagulation therapy, acquired von Willebrand factor deficiency, and GI arteriovenous malformations (53, 54). Recurrent GI bleeding events inevitably result in repeated hospital readmissions, impair patients' quality of life (QoL), and exacerbate long-term healthcare economic burdens. Accordingly, the QoL benefits conferred by LVAD implantation should be interpreted prudently from a balanced perspective. Although durable LVADs effectively ameliorate cardiac function and exercise tolerance in patients with end-stage HF, such functional improvements are substantially counterbalanced by recurring adverse complications, lifelong requirement for routine clinical surveillance, and heavy caregiving burdens (55). Overall, post-LVAD QoL status reflects a dynamic trade-off between enhanced physical functional capacity and persistent clinical challenges, including recurrent adverse events, continuous surveillance reliance, and dependence on caregiver support.

## 9 Future directions

Growing population aging and rising prevalence of advanced HF drive an increasing clinical demand for temporary and durable MCSDs (Figure 1). Future advances in MCSDs rely on not only technical and engineering innovations, but also refined patient selection, equitable device access, and rigorous clinical evidence generation (3, 18). Low- and middle-income settings still face substantial barriers to widespread MCSDs adoption, including inadequate infrastructure, high device costs, insufficient reimbursement, and limited long-term outpatient support. Therefore, healthcare system feasibility should be prioritized alongside device technical performance to ensure sustainable clinical implementation.

**Figure 1:**
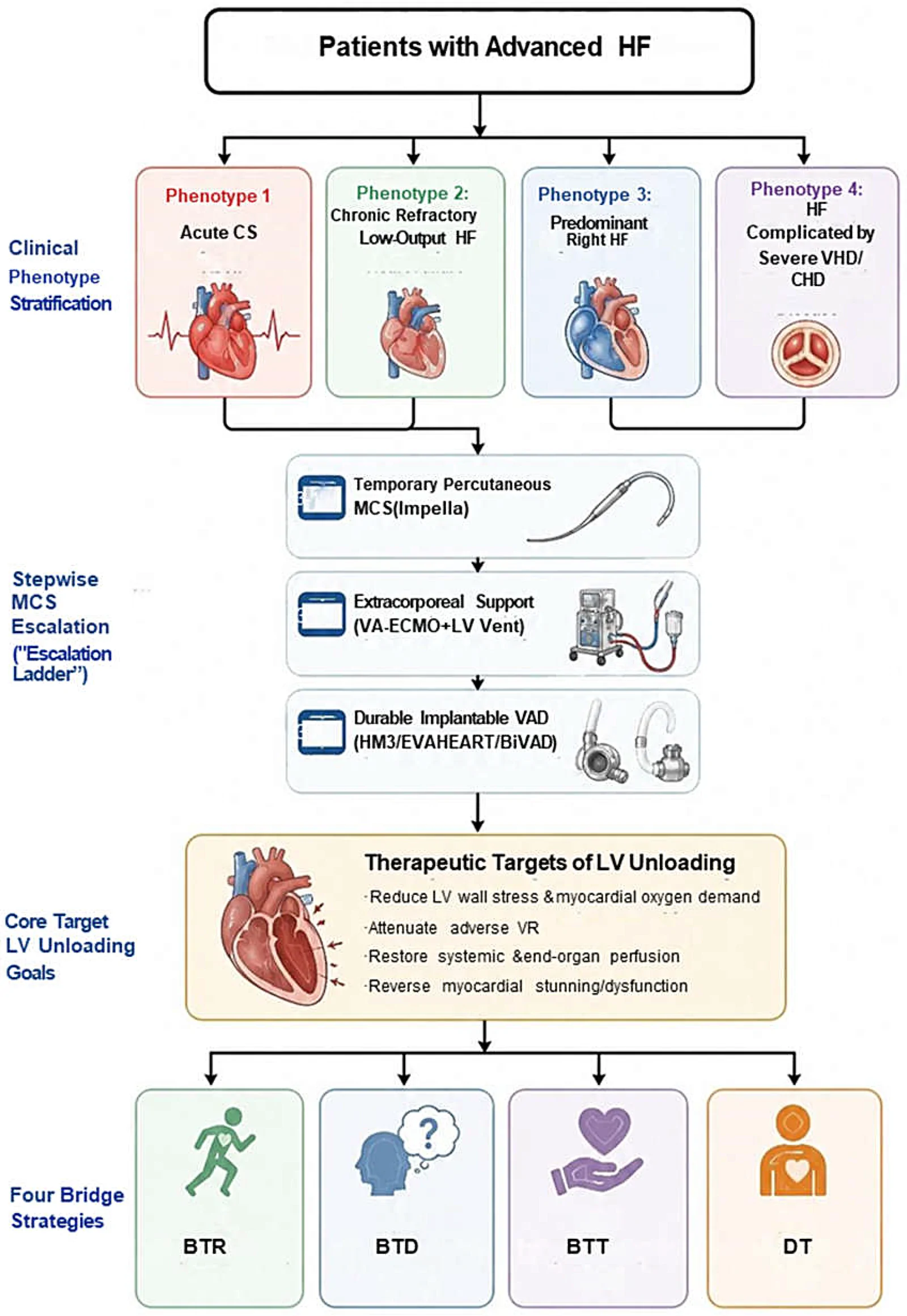
This figure centers on patient heart failure phenotypes and clearly visualizes core clinical workflows: individualized device selection, stepwise MCSDs therapy escalation, LV unloading strategies, and multi-modal bridge pathways (BTR, BTD, BTT and DT). HF, Heart failure; CS, Cardiogenic shock; VHD, Valvular heart disease; CHD, Congenital heart disease; MCSDs, Mechanical circulatory support devices; VA-ECMO, Veno-arterial extracorporeal membrane oxygenation; VAD, Ventricular assist device; LV, Left ventricle; VR, Ventricular remodeling; BTR, Bridge to recovery; BTD, Bridge to decision;BTT, Bridge to transplant therapy; DT, Destination therapy.

With the increasing burden of cardiovascular diseases and continuous technological iteration, MCSDs are expected to achieve expanded clinical application and improved therapeutic efficacy. The research and development of MCSD technology will likely focus on several directions (Figure 2). To avoid generalized descriptions and clarify the evolutionary roadmap of MCSD innovation, future developments can be systematically categorized into near-term, medium-term, and long-term priorities.

**Figure 2:**
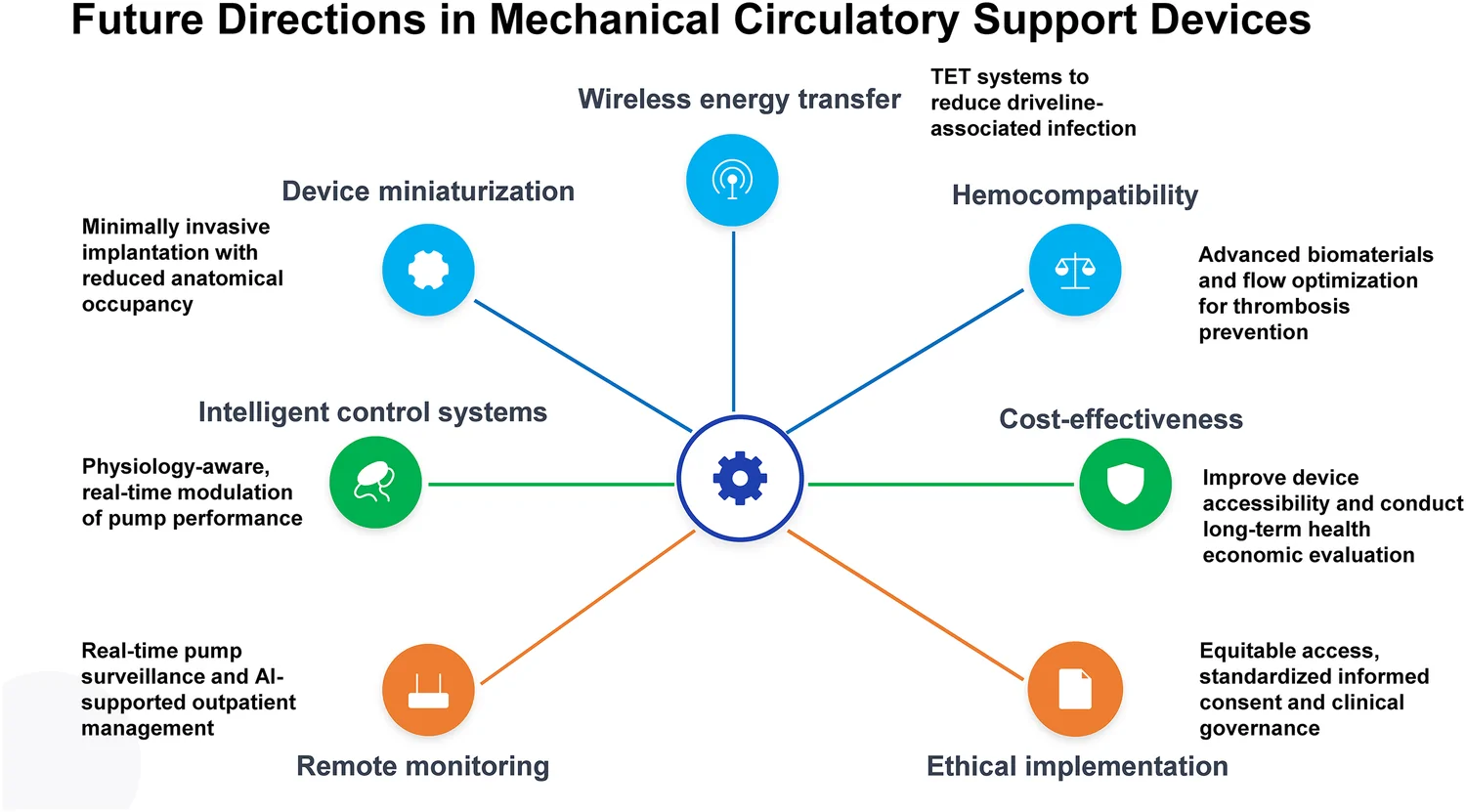
Schematic framework outlining seven core future development directions for MCSDs, covering hardware engineering optimization (device miniaturization, wireless energy transfer, hemocompatibility), intelligent digital control (intelligent control systems, cost-effectiveness), and clinical translation and regulation (remote monitoring, ethical implementation). MCSDs: Mechanical circulatory support devices; TET: Transcutaneous energy transfer; AI: Artificial intelligence.

### 9.1 Near-term developments

Near-term developments focus on iterative optimization of existing MCSD systems and clinical management strategies (18, 56). Current engineering improvements prioritize enhanced hemocompatibility to reduce common adverse events including hemolysis, stroke, and device-related infection, while strengthening device durability and operational stability. Clinically, standardized patient selection criteria and registry-based outcome reporting will be further optimized to screen eligible recipients precisely and accumulate high-quality real-world evidence for refining postoperative management and long-term prognosis.

### 9.2 Medium-term innovations

Medium-term innovations will focus on intelligent and minimally invasive upgrades to improve individualized therapy and patient experience (57). Remote physiological monitoring systems will enable real-time postoperative surveillance and early complication intervention. Optimized adaptive pump algorithms can dynamically adjust circulatory support parameters to match individual physiological conditions. Additionally, advanced minimally invasive implantation techniques will reduce surgical trauma and perioperative risks, broadening the clinical applicability of MCSDs therapy.

### 9.3 Long-term technological breakthroughs

Long-term technological breakthroughs aim to achieve transformative, fully integrated MCSDs systems (58). The clinical translation of fully implantable devices and wireless energy transfer technology will eliminate driveline-associated complications and substantially improve patients' mobility and QoL. Furthermore, artificial intelligence (AI)-empowered control systems integrating multi-sensor data, remote telemonitoring, and machine learning algorithms will realize automatic, personalized circulatory regulation, enabling precise treatment for complex advanced cardiovascular disease scenarios (59).

Beyond technological optimization, future MCSDs research should expand evaluation endpoints beyond overall survival, covering functional recovery, quality-adjusted life years, caregiver burden, and long-term cost-effectiveness (56). Ethical considerations remain indispensable for clinical decision-making, as technical feasibility does not justify MCSDs implantation in frail patients with irreversible multiorgan failure or minimal potential for functional recovery.

In conclusion, MCSDs hold promising prospects for the treatment of advanced cardiovascular diseases. To achieve high-quality and standardized development, the field should shift from pure technology-driven expansion to evidence-based, stage-specific, and patient-centered clinical implementation, maximizing long-term therapeutic benefits for eligible patients.

## 10 Conclusion

MCSDs have become an indispensable component of care for refractory CS and advanced HF. Temporary devices, durable LVADs, and TAHs each occupy distinct but overlapping roles, and their use should be guided by shock severity, INTERMACS profile, ventricular physiology, end-organ function, frailty, and the intended bridge strategy. The strongest contemporary evidence supports durable magnetic-levitation LVADs and structured stage-based selection, whereas many comparative questions for temporary support remain unresolved. Future progress will depend on better comparative trials, registry-quality reporting, complication reduction, and integration of ethical, economic, and health-system considerations into clinical decision-making.
