# Supplementary material for: Mechanical circulatory support devices in heart failure: a narrative review of current evidence, clinical applications, complications, and future directions
Source: Front Cardiovasc Med. 2026 Sep 29;13:1859170. doi: 10.3389/fcvm.2026.1859170 (PMC13658950; doi:10.3389/fcvm.2026.1859170)
Supplement: Supplementary file 1 [file Table1.docx]

Supplementary table 1. The search strategy and selection criteria

| **Serial No.** | **Category Description** | **Detailed Standardized Content (Fully Reproducible Methodological Details)** |
| --- | --- | --- |
| 1 | Full database-specific search strategy & complete Boolean search strings | 1. Databases included: PubMed/MEDLINE, Embase, Web of Science, Cochrane Library, CNKI, Wanfang, VIP  2. Separated full replicable Boolean search equations provided for each database; search fields: MeSH/Emtree subject terms, Title/Abstract/Keywords; complete Boolean operators (AND/OR/NOT) fully listed  3. Last search date recorded for every database; two-tier publication time filters defined: primary screening (2000.01.01–2026.03.31), snowball supplementary retrieval (pre-2000 landmark studies only)  4. Uniform limits: language (English / Chinese only), population (human adults ≥18 years), publication type limits (clinical trials, registries, cohorts, consensus guidelines, systematic reviews; exclude abstract-only records) |
| 2 | Inclusion & exclusion criteria | Inclusion: Full-text English/Chinese adult human clinical trials, multicenter registries, long-term cohort studies, clinical consensus guidelines, systematic reviews/meta-analyses focusing on MCSDs clinical application, device-related complications and technical innovations; pre-2000 landmark pioneering studies establishing foundational MCSDs clinical standards via snowball reference tracking  Exclusion: Animal experimental studies, single isolated case reports, duplicate overlapping articles, pure engineering in vitro mechanical manuscripts, conference abstract-only publications, pediatric patient research, non-clinical basic science papers |
| 3 | Hierarchical sorting criteria for overlapping guidelines, registries and reviews | Standard evidence priority ranking to resolve duplicate/overlapping literature:  RCTs > multinational multicenter registries > national real-world registries > long-term single-center cohorts > small observational studies > published systematic reviews/meta-analyses  Selection rules: Latest consensus guidelines and recent meta-analyses prioritized for overlapping review literature; manufacturer industry reports only used as supplementary non-peer-reviewed supporting data |
| 4 | Literature screening & formal quality appraisal workflow | Two independent trained reviewers performed two-stage screening (title/abstract screening → full-text screening); inconsistent screening results resolved by group discussion; remaining disputes adjudicated by a senior cardiologist third arbitrator  Uniform quality and bias appraisal standards applied to all English and Chinese included articles; identical eligibility and risk assessment criteria for domestic and overseas literature |
| 5 | Chinese literature identification and evaluation | Three major Chinese databases (CNKI, Wanfang, VIP) searched with unified standardized Chinese medical subject terms matching the English search framework; manual supplementary retrieval of domestic core cardiovascular journals and national heart failure/MCSDs clinical guidelines  Consistent screening thresholds and quality appraisal standards as English publications, no differentiated inclusion criteria |
| 6 | Pre-2000 landmark study selection rules | Snowball reference tracking from modern international and domestic MCSDs guidelines & classic comprehensive reviews; only retain pioneering pre-2000 studies establishing core clinical MCSDs practice standards; discard non-landmark early small observational studies without foundational clinical value |
| 7 | Full item-by-item SANRA compliance checklist | All mandatory SANRA core items explicitly documented with compliance status recorded one by one:  1. Prospective review protocol conceptualization  2. Comprehensive transparent literature retrieval strategy (full search strings, time limits, database records)  3. Predefined standardized inclusion/exclusion criteria  4. Formal dual-reviewer screening and conflict resolution mechanism  5. Standardized study quality and bias risk evaluation  6. Clear hierarchical evidence grading framework  7. Balanced unbiased narrative synthesis without selective reporting  8. Complete disclosure of literature limitations and evidence heterogeneity  All methodological SANRA indicators followed per structured narrative review characteristics, with full traceable documentation |
